# Retinoid-X receptor action and disruption in development

**DOI:** 10.1016/j.ydbio.2026.03.003

**Published:** 2026-03-10

**Authors:** Brenda J. Mengeling, J. David Furlow

**Affiliations:** Department of Neurobiology, Physiology, and Behavior, College of Biological Sciences, University of California Davis, Davis, CA, USA

## Abstract

Across the animal kingdom, many developmental programs require retinoid X receptor (RXR) expression. RXR is a unique member of the nuclear receptor (NRs) superfamily of transcription factors. Most famously, it serves as a mandatory heterodimer partner for a third of the other vertebrate NRs, such as retinoic acid receptors (RARs), thyroid hormone receptors (TRs), the vitamin D receptor (VDR), and peroxisome proliferator-activated receptors (PPARs), which all have important roles in development. RXR is also one of the most ancient NRs, with expression in most metazoan lineages going back to basal metazoans such as placozoans and cnidarians, and we review examples across the major animal phyla of RXRs diverse roles in animal development, especially in control of metamorphic genetic programs. We also review the open area of RXR research that seeks to determine the endogenous ligands of RXR and when and where they are required for RXR action. Further, we discuss the current understanding from cellular data with implications for development of how organisms may control the availability of RXR for NR partnership and the hierarchy of NR access to a limited RXR pool. Finally, we will review the ability of certain man-made chemicals in the environment to disrupt RXR signaling during development.

## 1. Introduction and mechanisms of action

Across the animal kingdom, the roles of nuclear receptors (NRs) in development are difficult to overstate. And retinoid X receptors (RXRs) are central to NR action because many NRs involved in development require heterodimerization with an RXR to become a functioning transcription factor. For example, in chordates, the RXR-RAR (retinoic acid receptor) controls patterning and organogenesis (Chawla et al., 2016; Cunningham and Duester, 2015; Nedelec et al., 2019; Williams and Bohnsack, 2019); the RXR-TR (thyroid hormone receptor) controls central nervous system, long bone and gut maturation, and metamorphosis (Buchholz, 2017; Das et al., 2009; Furlow and Neff, 2006; Laudet, 2011; Wang et al., 2008); the RXR-VDR (Vitamin D receptor) controls lung maturation and immune system development (Arora et al., 2024; Foong et al., 2015; Mandell et al., 2020); and RXR-PPARγ (peroxisome proliferation-activated receptor) programs adipocyte development (Barak et al., 1999; Rosen et al., 1999; Tontonoz et al., 1994).

**Fig. 1.**
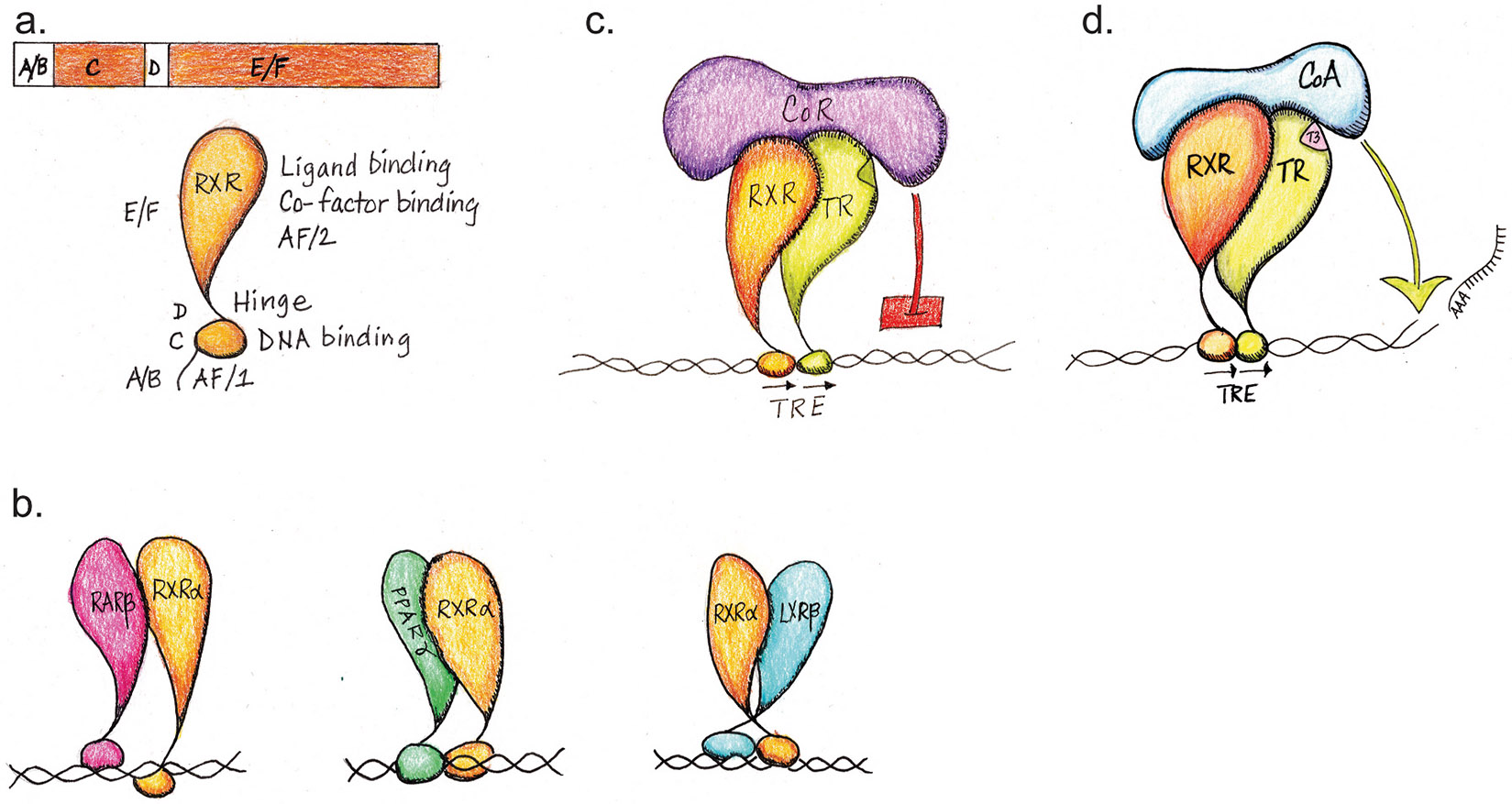
RXR structural and activity characteristics. A. RXR shares its domain structure with other members of the nuclear receptor superfamily of transcription factors. B. In heterodimerization, RXR provides flexibility to accommodate its binding partners and their DNA-binding recognition sequences. C. Using the TR-RXR heterodimer as an example, in the absence of T3, the heterodimer binds corepressor, which creates a scaffold for activities that produce a chromatin landscape refractory to transcriptional activation. D. Upon TR binding T3, corepressor is dismissed and coactivator binds, which creates a chromatin landscape favorable to transcriptional activation.

The role of RXR, especially in development, did not begin with chordates. RXRs are one of the most ancient NRs, recognizing that NRs are only found in the animal kingdom. Placozoans, possibly the most basal metazoan animal still extant (see below) expresses four NRs: HNF4A, ERR, COUP-TF, and RXR. All NRs from placozoans through humans share a common domain structure (Fig. 1A) composed of domains A-F in order from amino-terminus to carboxy-terminus. The C-domain or DNA-binding domain (DBD) shows the highest conservation across phyla and consists of two zinc-finger domains which interact with the DNA and some dimerization surfaces. The next highest conserved domain is the E-domain or ligand-binding domain (LBD). The LBDs share an overall structure with eleven alpha helixes creating a threelayered sandwich that, in addition to binding ligand, interacts with coregulator proteins, has dimerization interfaces, and an AF/2 (activating function 2) helix (helix 12) required for ligand-induced transcriptional activation (Bourguet et al., 2000; Lou et al., 2014; Rastinejad, 2022; Tambones and Le Maire, 2025). The hinge, D-domain connects the C and E domains, and is variable among NRs. This variability is maintained in the A/B-domain, which is unstructured in most NRs and may contain an AF/1 sequence, and the F-domain that follows helix 12 of the LBD; not all NRs, including RXRs, have a substantial F-domain.

Type II NRs form heterodimers with RXRs (Lefebvre et al., 2010). Heterodimerization facilitates or is required for DNA binding (Chandra et al., 2008; Gampe et al., 2000; Kliewer et al., 1992; Puzianowska--Kuznicka et al., 1997; Willy et al., 1995; Willy and Mangelsdorf, 1997; Yen et al., 1992). The partner of the RXR dictates the NR-RXR DNA binding site. Essentially, the two proteins bind to a direct repeat of AGGTCA with an NR-defined spacing between the two repeats (e.g. the TR-RXR heterodimer preferably binds to a DR4 element, where a 4 nucleotide-spacer lies between to two binding sites). X-ray crystallography studies of full-length NR-RXR heterodimers demonstrate that the RXR is a flexible partner, allowing for the different spacing of the direct repeat binding sites for each NR-RXR pair (Fig. 1B) (Chandra et al., 2008, 2017; Lou et al., 2014; Rastinejad, 2022). Type II NRs reside in the nucleus even in the absence of agonist ligand. In the absence of ligand, the heterodimer binds a corepressor protein (Fig. 1C), which on genes activated by the receptor in the presence of ligand, are now repressed below basal (i.e. in the absence of the NR) levels. The corepressor functions as a scaffold for binding proteins involved in making chromatin inaccessible to the Mediator and RNA polymerase complexes, such as histone deacetylases (HDACs). Upon binding ligand, the conformation of the NR is changed, dismissing the corepressor and allowing the binding of coactivators (Fig. 1D), which have histone acetyltransferase activity, which in turn increases the accessibility of the chromatin to the transcriptional machinery.

The role of the RXR and its ligands (see below) in any heterodimer depends on the NR partner. For what have been traditionally known as “permissive” partners of RXRs, the RXR ligand can activate the heterodimer even in the absence of the ligand for the other NR partner, and when both ligands are present, the activation is synergistic compared to either ligand alone. PPARs and LXRs are hallmarks of this type of regulation (Kliewer et al., 1992; Willy et al., 1995; Willy and Mangelsdorf, 1997). “Non-permissive” partners do not allow the RXR ligand to activate transcription in the absence of the partner ligand. Traditionally, TRs, RARs, and VDR fall into this category, although there are now exceptions. For example, in cells where the ratio of coactivator to corepressor is high, RXR agonists can affect TR action (Castillo et al., 2004; Mengeling and Furlow, 2015). In vitro and cellular work suggested that RXR agonists can, in the absence of TH, dismiss corepressor, while not inducing coactivator binding (Fattori et al., 2015) Structural and functional studies of the RAR-RXR heterodimer used ligands for each receptor that control corepressor or coactivator binding to the heterodimer. The studies suggested that agonist-bound RAR could dismiss corepressor, and then coactivator could bind through agonist-bound RXR even in the absence of an RAR agonist. However, synergistic activation occurred when both receptors bound agonists (Cordeiro et al., 2019; Germain et al., 2021; Le Maire et al., 2019). The Aranda lab found that VDR reporter and endogenous gene activation could be potentiated by the presence of 9-cis retinoic acid (9c-RA), an endogenous agonist of RXR, including increasing the activation by a VDR partial-agonist and by a mutant VDR that causes hereditary rickets; RXR agonism also increased the ability of vitamin D to induce differentiation of a colon cancer cell line (Sánchez-Martínez et al., 2006). In addition, Doost et al., found synergistic activation of VDR signaling in the presence of RXR designed agonists, and that the ability of the RXR agonist to synergize with the VDR depended on the chemical class of the RXR agonist (Doost et al., 2024). Thus, RXR participation in both permissive or non-permissive dimerization appears affected by coactivator and corepressor expression levels, the nature and order of addition of the cognate ligands, and may be gene specific.

### 1.1. RXR tetramers and availability

**Fig. 2.**
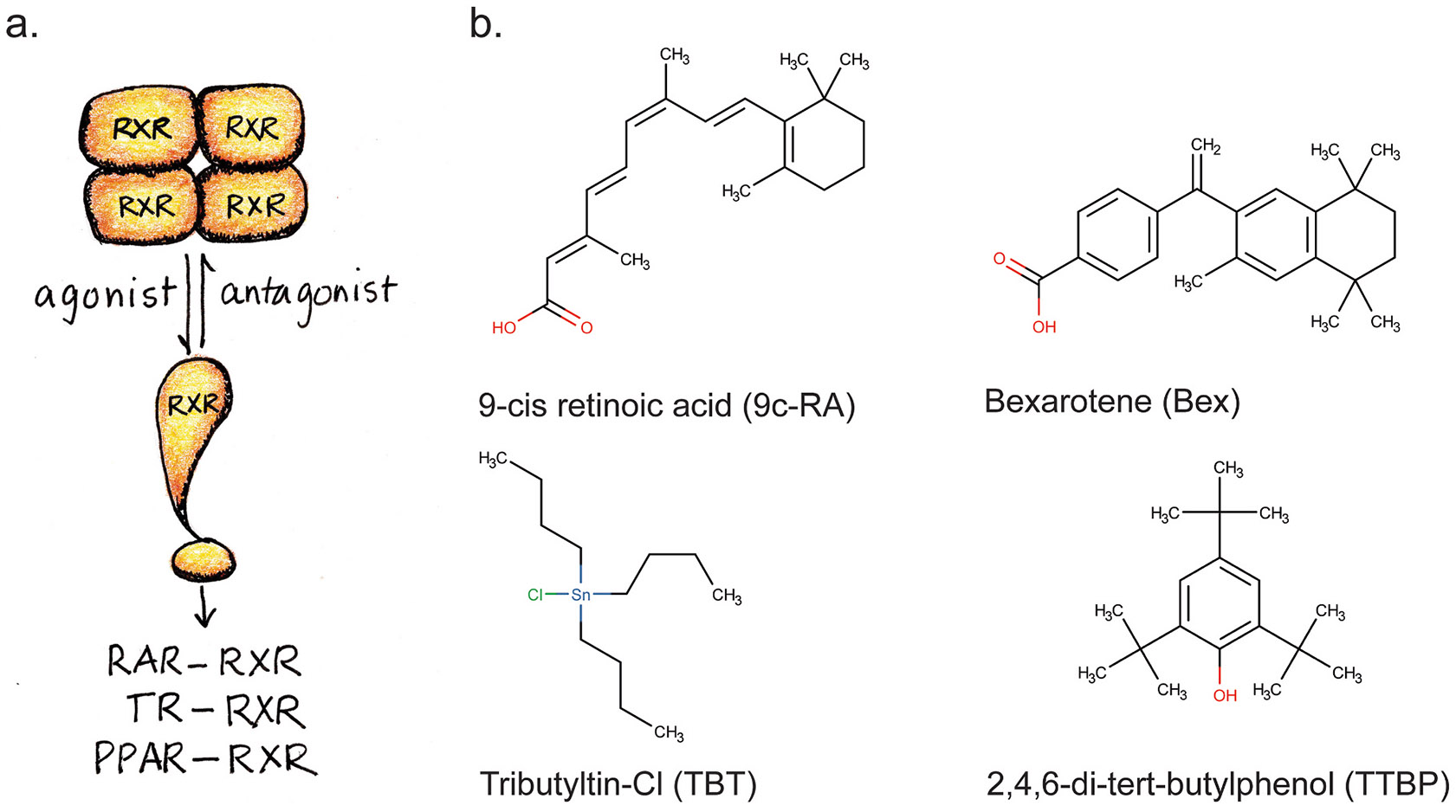
RXR ligands may affect its availability for heterodimerization with binding partners. A. In solution, RXRs, in the absence of ligand or in the presence of antagonist, form tetramers that appear inactive and unavailable to binding partners. Upon binding agonist, the RXR tetramers dissociate into monomers available for heterodimerization. B. Structures of RXR agonists: 9-cis retinoic acid is an endogenous agonist, bexarotene a pharmaceutical agonist, and tributyltin and 2,4,6-di-tert-butylphenol are environmental pollutants that have been shown to be able to function as RXR agonists.

Unique among NRs, RXR in the absence of ligand forms stable, tetramers in solution (Kersten et al., 1995). The tetramer cannot activate transcription in this state, as the helix 12 (AF/2 region) of one subunit blocks the coactivator binding site of the neighboring subunit (Zhang et al., 2011a). Upon agonist binding, the tetramer dissociates into homodimers and monomers (Chen et al., 1998; Dong and Noy, 1998). The homodimers can bind RXR-specific response elements and induce transcription (Fig. 2A). However, in the absence of the cognate hormone for a heterodimer partner, the RXR did not form heterodimers with tested NRs (RAR and VDR) (Dong and Noy, 1998). This mode of action, which has been studied mostly in vitro with purified proteins, has been suggested to be a mechanism for controlling the amount of RXR available to binding partners.

Two single amino acid mutants of the mouse RXRα were made that either maintained the RXR in a tetramer even in the presence of RXR agonist (R321A) or were able to homo- and hetero-dimerize but not form tetramers (F318A) (Kersten et al., 1998). Tested in a reporter assay in HeLa cells, the R321A was strongly compromised in its ability to activate 9-cis retinoic acid (9c-RA)-induced reporter gene expression; whereas the F318A mutant, which couldn't form tetramers, showed higher levels of reporter gene activation compared to wildtype RXR even in the absence of 9c-RA.

### 1.2. Competition for RXR

All mammalian cells express at least one of the three RXRs, and most express multiple NR binding partners for RXR. Is RXR the limiting factor in NR heterodimerization? Usually, this is assumed to be the case due to expression of multiple NRs wanting to bind to RXR and the ability of RXR to form homotetramers in solution. Recently, the Darzacq lab used genome editing to tag the endogenous genes for RAR and RXR in U2OS cells, ensuring that normal levels of the two NRs were maintained. Using single-molecule tracking and proximity-assisted photoactivation methods, they found that increasing the expression of RAR but not RXR increased heterodimerization of RAR to RXR (Dahal et al., 2025). They equated expression with bioavailability, which research on RXR tetramers (see above) and ChIP-seq experiments suggest may not be assumed. For example, addition of RXR agonists increases the ChIP-seq signal at binding sites bound in the absence of RXR agonist (Daniel et al., 2014), rather than increasing or changing the repertoire of sites bound by the heterodimer.

Is there a hierarchy of which NR partner gets access to the available RXR first? Using the human breast cancer cell line T47D, which endogenously express VDR at high levels, the Mehta lab showed that PPARγ expression inhibited the ability of the natural agonist for the VDR, calcitriol, to activate a VDR-driven luciferase reporter, especially when they increased the expression of RXRα, suggesting that PPARγ outcompeted the VDR for RXR dimerization (Alimirah et al., 2012). The Vamosi group compared the abilities of human RARα, VDR, and PPARγ to bind to RXRα when RXR expression was limiting (Fadel et al., 2020). Their cellular model used expression of the NR partners with either EGFP or mCherry fluorescent protein tags in pairs with BFP-RXR in HEK293 cells, which endogenously express low levels of NRs. They used RARα and PPARγ with mutations in the nuclear localization signal (NLS), which kept them in the cytosol unless they were able to bind to the RXR and use its NLS to enter the nucleus; VDR without mutation of its NLS remained mostly in the cytosol when BFP-RXR was not expressed and so was not mutated. Transit of the distinct NRs into the nucleus was then quantitatively assessed. In the absence of agonist for any partner NR, the hierarchy for dimerizing with the limiting RXR was RAR *>* PPAR *>* VDR. However, when any one agonist was present, that NR preferentially had access to the RXR pool. When NR partners were assayed in pairs with both of their agonists present, RARα outcompeted both PPARγ and VDR for RXR, and PPARγ out competed VDR. In a follow-up study, they compared the ability of VDR and RAR to compete for DNA binding in cells (Rehó et al., 2023). They expressed RAR, VDR and RXR together in cells, with distinct fluorescent tags, and they measured the diffusion constants of the different heterodimers on a selected plane illumination microscope. A slower diffusion constant indicated DNA binding, and it was assumed that DNA binding only occurred from heterodimers. RAR again outcompeted VDR for RXR heterodimerization when the cells were treated with agonists for both NRs. Interestingly, addition of the synthetic RXR agonist LG268 to the above experiment increased the ability of RAR-RXR to form and bind DNA but decreased the ability of VDR-RXR to form and bind DNA (Rehó et al., 2023).

### 1.3. RXR ligands

Mammalian RXRs bind a diverse set of chemicals that have been shown to activate RXR-induced reporter assays or endogenous gene expression. RXR ligands include endogenous compounds (Heyman et al., 1992; Kitareewan et al., 1996; Lengqvist et al., 2004; Rühl et al., 2015), pharmaceuticals (Boehm et al., 1995; Leibowitz et al., 2006; Michellys et al., 2003; Spathis et al., 2017; X. Yu et al., 2023), environmental compounds (Kotani et al., 2010, 2012; Scheepstra et al., 2014; Zhang et al., 2011b; Zhang et al., 2011c; Zhang et al., 2011d), and man-made chemicals that inadvertently disrupt RXR action (Harmon et al., 1995; le Maire et al., 2009; Mengeling et al., 2018, 2025; Ren et al., 2023). They have been reviewed extensively elsewhere (Dawson and Xia, 2012; Krężel et al., 2019; Pérez et al., 2012). Fig. 2B shows an example compound from each category of RXR agonist.

RXR liganding has most often been studied in conjunction with an NR partner's activity; however, three vertebrate reporter animal models have shown in vivo activation of RXR during development by endogenous ligands. In transgenic *Xenopus laevis*, Luria and Furlow expressed the LBD of RXRα fused to the DNA-binding domain of the yeast Gal4 transcription factor (Gal4-DBD) in conjunction with a Gal4-UAS (upstream-activating sequence) array-controlled enhanced green fluorescent protein (EGFP) reporter construct. The reporter gene was activated in the spinal cord immediately posterior to the hindbrain starting at the neurula stage through the swimming tadpole, at which time the signal declined to background levels (Luria and Furlow, 2004)An earlier study from the Perlmann laboratory showed a remarkably similar pattern of RXR LBD-Gal4 activation in the developing mouse anterior spinal cord (Solomin et al., 1998). In addition, the Welch lab used this reporter strategy to search for endogenous RXR ligands in hematopoietic cells. They found that reporter activity was stimulated by granulopoiesis and by drug-induced anemia. Interestingly, making the mice vitamin A deficient did not cause a decrease in reporter activation; however, a diet restricting fatty acids did cause a decrease in reporter activation, and they identified C24:5 long-chain fatty acid as a natural RXR agonist in mouse hematopoietic cells (Niu et al., 2017).

The most often cited endogenous RXR ligand has been 9c-RA, which activates the receptor at low nanomolar concentrations (Allenby et al., 1993; Heyman,’ et al., 1992; Levin et al., 1992). However, due to often undetectable levels of 9c-RA in mammalian embryos and adult tissues, acceptance of 9c-RA as the predominant endogenous ligand is controversial (Kane, 2012; Wolf, 2006). Nevertheless, X-ray crystallography has revealed 9c-RA to be bound in the LBP of RXR (Egea et al., 2000), including in a crystal of full-length PPARγ-RXRα heterodimer bound to DNA (Chandra et al., 2008). The RXR LBP also requires a bend in the ligand that 9c-RA has and all-trans retinoic acid (ATRA), the predominant retinoic acid found in cells, does not (Pérez et al., 2012).

Independent of whether 9c-RA is the *bona fide* endogenous ligand for mammalian RXRs, in determining whether an action is RXR regulated, or whether a natural environmental or man-made chemical is an RXR ligand, comparison to 9c-RA-induced or RXR pharmaceutical induction is the standard to meet. And in surveying RXR action across invertebrate animal phyla, 9c-RA is often an activator, and in cases where endogenous levels were measured, it was found at reasonable concentrations for its affinity for the receptor (see below).

## 2. Examples of RXR action in development across the animal kingdom

**Fig. 3.**
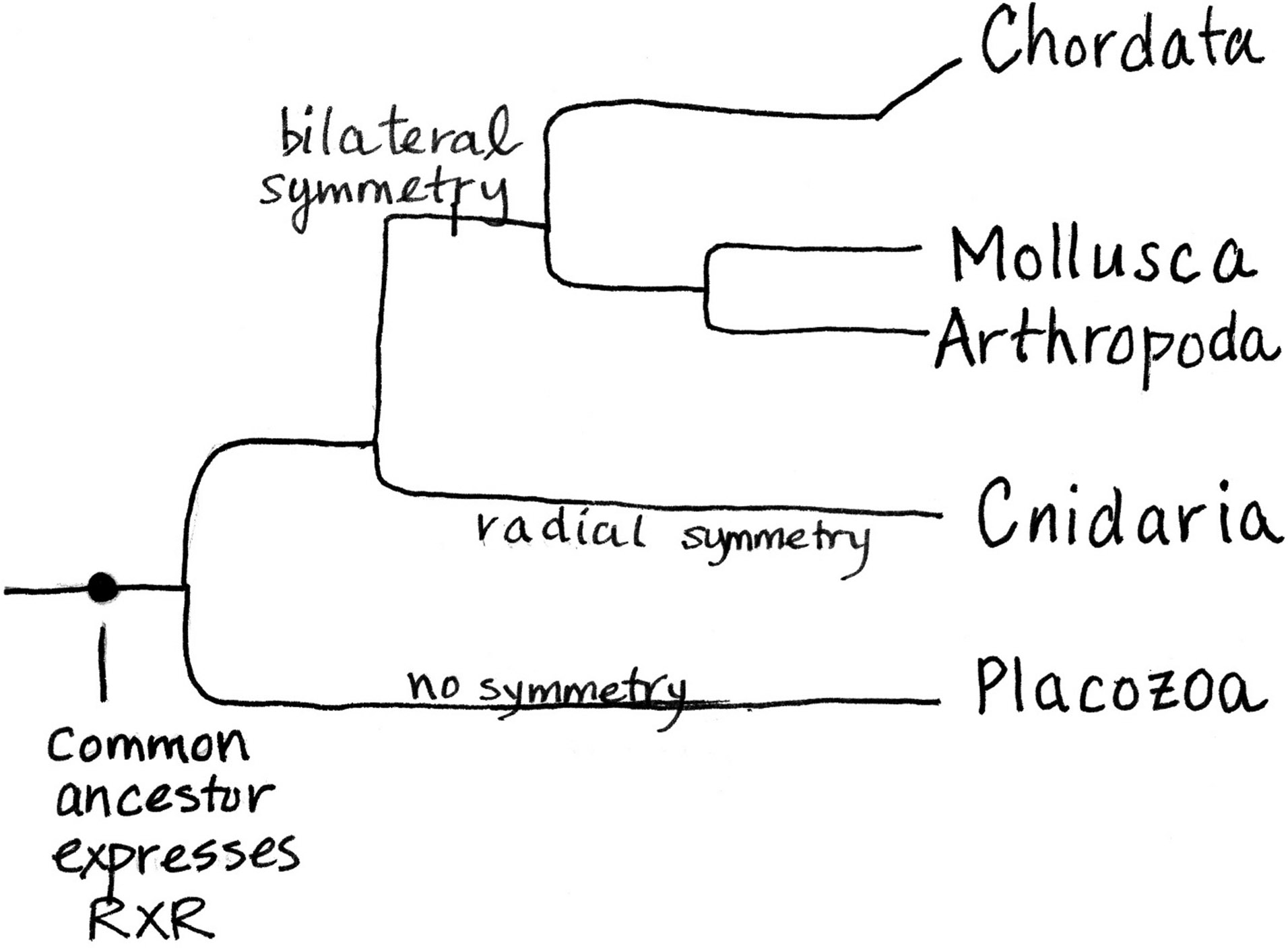
Phylogenetic relationships and organismal symmetry between animal phyla discussed. Note that of the placozoans, only sponges appear to lack an RXR homolog. Further, in chordates, two rounds of genome duplication led to three distinct RXR encoding genes that have evolved overlapping and distinct functions in vertebrates.

Today, even in the simplest metazoan animals RXR is expressed, which suggests that NRs and RXRs evolved in a common non-extant ancestor. Here we discuss RXRs and development in five distinct animal phyla, where there the role of RXR has been explicitly studied. Fig. 3 shows these five phyla and there relatedness in terms of organismal symmetry. More detail on the evolution of RXR can be found in the following (Fonseca et al., 2020; Hult et al., 2011; Mottes et al., 2021).

### 2.1. Placozoans

Placozoans are among the simplest of metazoan animals, and they have become a model for studying the evolution of animal cell types (Schierwater and DeSalle, 2018). They have no symmetry, no neurons or muscles, no tissue organization, and they are comprised of only a half dozen or so cell types with a simple dorsal-ventral polarity (Romanova and Moroz, 2024). The genomes of two species (*Trichoplax adhaerens* and *Hoilungia hongkongensis*) have been determined (Eitel et al., 2018; Srivastava et al., 2008), and both genomes contain an RXR gene. Phylogenetic analysis of Porifera (sponges), another ancient metazoan animal phylum, failed to detect a definitive RXR homolog (Reitzel et al., 2018). The *T. adhaerens* RXR gene has been studied, and it shows remarkable identity to human RXRα at the amino acid level of 81% identity in the DNA binding domain and 70% in the ligand binding domain (Novotný et al., 2017). In vitro, competitive binding assays showed that *T. adhaerens* RXR binds 9c-RA but not ATRA with low nanomolar affinity (saturation at 5-10 nM 9c-RA). Further, 1-3 nM 9c-RA induced expression of the metabolic gene L-malate-NADP + oxioreductase, which is controlled in humans by TR-RXR. Reitzel et al. used a protein-binding microarray to compare the DNA sequences preferred by *T. adhaerens* RXR compared to human RXRα, and they found an 85% overlap in DNA recognition sequences, indicating that the DNA binding properties of RXR were established and then maintained over a billion years of evolution (Reitzel et al., 2018). Developmentally, culturing *T. adhaerens* in presence of 3.3 nM 9c-RA with phycobilin-expressing red algal food source, resulted in a significant decrease in the total area and perimeter of the organism over a 24 h period; however, 9c-RA had no significant effect on *T. adhaerens* fed a green algal food source (Novotný et al., 2017).

### 2.2. Cnidarians

Cnidarians have a more complex body plan than placozoans, being diploblasts, having radial symmetry, and a neural net. In terms of RXR expression, there has been the intriguing finding that cnidarians with a medusa (jellyfish) stage in their lifecycle have *bona fide* RXR genes (Fuchs et al., 2014; Kostrouch et al., 1998), and those without one, like the hydra, sea anemones, and corals, do not appear to have an RXR gene (Chapman et al., 2010; Putnam et al., 2007; Shinzato et al., 2011). *Tripedalia cystophora* are one of the most ancient, extant species of jellyfish, and they possess an RXR gene with 61% amino acid identity in the DBD and 68% identity in the LBD to human RXRα. It binds 9c-RA with a 0.4 nM kd, and when expressed in vitro could dimerize with *Xenopus laevis* (African clawed frog) TRβ, and that heterodimer could bind to a TR-specific DNA response element (Kostrouch et al., 1998). This study did not determine whether the *T. cystophora* produce 9c-RA, and it did not examine expression over developmental time.

The jellyfish lifecycle comprises several different stages. Sexually mature pelagic medusae produce egg and sperm that after fertilization develop into a ciliated planula that becomes a sessile polyp. The polyp develops into a strobila through a metamorphic process called strobilation wherein a series of disk-like segments form. Each segment becomes an ephyra (young jellyfish), which matures into an adult, reproductive medusa, which is what we picture when we hear the term “jellyfish.” For the moon jellyfish *Aurelia aurita*, seasonal cooling of the water temperature provides the environmental cue to begin strobilation, which produces young jellyfish by spring and early summer. Fuchs et al. took advantage of the ability to induce strobilation in a laboratory setting by changing the water temperature from 18 °C to 10 °C to examine the molecular cascades the temperature change initiated (Fuchs et al., 2014). They compared the transcriptomes of the polyp, strobila, and ephyra stages, and they noted that the *A. aurita RxR* gene, along with the *RDH2* gene, which oxidizes retinol to retinaldehyde in the path to retinoic acids, were induced during strobilation. In fact, they were able to induce strobilation without lowering the water temperature (i.e. maintaining it at 18 °C) by including 1 μM retinol or 9c-RA in the water. In addition, inclusion of the mammalian RXR antagonist UVI3003 inhibited strobilation, even when the animals were switched to water at 10 °C (Fuchs et al., 2014). Transcriptomic analysis of differential gene expression over developmental time in the jellyfish *Rhopilema esculentum* also determined that *RxR* expression rose dramatically during strobilation and then decreased sharply in the ephyra (Ge et al., 2018).

### 2.3. Arthropods

Over evolutionary time, the phylum Arthropoda has contributed one of the largest changes to RXR in the form of its homolog Ultraspiracle (USP). In all arthropods, USP/RXR functions as the heterodimer partner to the ecdysone receptor (EcR). Ecdysone signaling initiates molting and metamorphosis in larvae or nymphs. Basal insects like *Locusta migratoria* express an RXR that is more similar to vertebrate RXRs than the USP of later evolved orders like Lepidoptera (butterflies and moths) and Diptera (two-winged flies) (Hayward et al., 1999). *L. migratoria* embryos had measurable nanomolar levels of 9c-RA, and competition binding assays showed that its RXR bound 9c-RA with a similar IC-50 to human RXRα (Nowickyj et al., 2008). Locusts are hemimetabolous insects which undergo a partial metamorphosis through a series of molts from an immature nymph to a reproductive adult. USP lost its ability to bind ligand between the evolution of Orthoptera (locusts) and Hemioptera (true bugs like aphids) and Coleoptera (beetles) (Iwema et al., 2007, 2009). Further evolution to Diptera and Lepidoptera re-created the ligand-binding pocket in the LBD of USP, but now phospholipids appear to be bound (Clayton et al., 2001). Jones et al. found that purified, bacterially expressed *Drosophila* USP can bind to farnesoid derivatives, including juvenile hormone (JH) with nanomolar efficiency (Jones et al., 2006). However, Jindra et al. showed in vivo that mutants of *Methoprene-tolerant* (Met) and its paralog *germ cell-expressed* (Gce) that prevented JH binding abrogated the ability of organism to respond to JH or designed JH mimics (Jindra et al., 2015), Holometabolous insects undergo a complete metamorphosis from a larval form (e.g. a caterpillar) through a pupa to an adult form (e.g. a butterfly). They evolved more recently than the hemimetabolous insects. The ligand-binding domain of USP in holometabolous insects underwent several changes that appear to be under positive selection (Hult et al., 2011). These changes create a larger dimerization surface with the EcR (Hult et al., 2011; Iwema et al., 2009).

The role of ecdysone signaling through EcR/USP during embryogenesis has primarily been studied in the holometabolous, long-germ band insect *Drosophila melanogaster*, where it is required for germ band retraction, head involution, and major tissue movements required to generate a first instar larva (Chavoshi et al., 2010; Kozlova and Thummel, 2003). *Blattella germanica*, the German cockroach, is a short-germ band, hemimetabolous insect, and the RXR it expresses is more similar to vertebrate RXRs than holometabolous USPs. Cruz et al. investigated the role of EcR/RXR in *B. germanica* during embryogenesis (Cruz et al., 2023). They used parental RNAi technology to silence the expression of either the BgEcR-A or BgRXR genes. Embryos developed normally for 30-36 h, but later time points showed a failure of the embryos to form the germinal band, which is the part of the embryo that becomes the insect body, due to significantly decreased cell proliferation; however, the knockdowns did not affect serosal cell fate, which appeared to develop normally. The germinal cells that did exist correctly migrated to the ventral side of the embryo forming a diminished germ band. In addition, the BgEcR-A and BgRXR knockdowns prevented the induction at the maternal-to-zygotic transition of other NRs required for embryogenesis. In particular, failure to induce the direct ecdysone responsive HR3-A gene resulted in the loss of head structures in the germ band (Cruz et al., 2023). This requirement for EcR/RXR signaling as early as the maternal-to-zygotic transition and the formation of the germ band in *B. germanica* is an earlier requirement than in *D. melanogaster* embryogenesis. The authors speculate that the loss of the requirement for EcR/RXR signaling so early in embryogenesis in long-band insects may have favored the evolution from short-germ band to long-germ band (Cruz et al., 2023).

### 2.4. Mollusks and the discovery of environmental disruption of RXR

Our understanding of RXRs in mollusks started with oyster beds in the Arcachon Bay on the Atlantic coast of France. In the 1970s Arcachon Bay was home to oyster farming and pleasure craft marinas, and oyster production crashed due to stunted growth rates, shell calcification anomalies, and a total loss of reproductive capacity. The organotin tributyltin (TBT), an antifoulant in the paint on the pleasure craft, was shown to be the causative agent (Alzieu, 1991, 2000). TBT induces imposex in female mollusks, which is the acquisition of male secondary sex characteristics, like penile and vas deferens development, rendering the female infertile (Horiguchi et al., 1997; Mensink et al., 2002; Urushitani et al., 2018). Furthermore, injection of 9c-RA into female mollusks induced imposex, and the mollusk RXR bound 9c-RA and showed the highest expression in genitalia, specifically male-type genitalia (Castro et al., 2007; Horiguchi et al., 2007; Nishikawa et al., 2004).

The Giulianelli lab collected two different species of marine gastropods (snails) from two different locations of the same Argentinean bay. One site had TBT contamination due to shipping traffic, and the other was relatively unpolluted. The two slipper snail species have different sensitivities to imposex development. *Trophon geversianus* (Pallas 1774) is resistant to imposex even when residing in TBT-contaminated harbors, while *Buccinanops globulosus* (now more properly *Buccinastrum deforme*) is susceptible to imposex development. The gastropod RXR proteins reacted specifically with anti-RXR antibodies derived against mammalian RXR, and the authors found that the resistant species, *T. geversianus*, expressed significantly lower levels of RXR in the smooth muscle cells surrounding the epithelium of the vas deferens than the imposex susceptible species. PPARγ levels, which are expressed in the same cells, did not differ between the two species. However, both males and imposex females of *B. deforme* from the TBT-polluted harbor water had significantly lower RXR-positive cells and expression levels than males of the same species from the unpolluted area of the bay (Giulianelli et al., 2020). Cloning of the RXR gene from each species showed 90% identity to human RXRα in the DBD, and 81% amino acid identity in the LBD (Giulianelli et al., 2025). The LBDs of both had the critical amino acids for binding to 9c-RA and the Cys to covalently bind to TBT (le Maire et al., 2009). Using LBDs of each RXR fused in frame to the yeast Gal4 DBD, the authors expressed them along with a Gal4-driven luciferase reporter plasmid in COS-1 cells, where both were significantly activated by 10 nM 9c-RA and 1 nM TBT. The LBD from the imposex susceptible species *B. deforme*, responded significantly to 0.1 nM TBT. The RXR antagonist UVI 3003 abrogated the TBT induction (Giulianelli et al., 2025).

These results raise the question of what RXR activation does in normal gastropod reproductive development. Gastropods that are sequentially hermaphroditic first become male, and when larger, develop into females through unknown molecular mechanisms. The Lesoway lab investigated male reproductive development by exposing juvenile *Crepidula astrosolea* slipper snails to ligands for RAR and RXR. Previous work had suggested that the gastropod RARs did not bind ATRA but that it functioned as an agonist for RXR in reporter assays (André et al., 2019). Both ATRA, TBT, and the vertebrate RAR agonist TTNPB could significantly initiate male genital development as assessed by penis length (Lesoway and Henry, 2021). Furthermore, RXR expression shifted from neural tissue adjacent to the site of penis development to the penile tissue itself, suggesting a role for RXR signaling in male genital development.

### 2.5. Vertebrate RXRs

At the origin of the vertebrate lineage, two rounds of whole genome duplication resulted in establishment of three RXR genes: RXRα, RXRβ, and RXRγ (Mottes et al., 2021). A third round of genome duplication led to additional paralogs for each of the RXR isotypes in zebrafish (Waxman and Yelon, 2007a). Presumably, as has been more thoroughly discussed for RARs (Campo-Paysaa et al., 2008, 2015; Escriva et al., 2006), this strategy allowed for preservation of the ancestral RXR functions with opportunity for expansion and/or fine tuning of roles for additional RXR encoding genes (Philip et al., 2012). The original discovery of RXRs was a convergent effort of a) searching the human genome for related sequences encoding additional retinoic acid receptors (Mangelsdorf et al., 1990) and b) strategies to identify mammalian factors that enhance NR binding to their response elements (Marks et al., 1992; V. C. Yu et al., 1991). The first nonmammalian RXRs were cloned from *Xenopus laevis* oocyte cDNA libraries (Blumberg et al., 1992; more about Xenopus RXRs will follow in the subsequent section), and subsequently all three RXR isotypes have been identified in all vertebrate species examined to date. The DNA and ligand binding domains of vertebrate RXRs are more conserved with their cognate isotypes across vertebrate species (nearly 100%) than across other RXR isotypes within the same species, although sequence identity still ranges between 85 and 90% in these domains (Mangelsdorf et al., 1992; Nagata et al., 1994). Most sequence diversity in RXR coding sequences is found in the amino terminal AF-1 domain which is a common feature of the NR superfamily as a whole, including the presence of alternative splicing or alternate translational start sites (Kojo et al., 2004; Nagata et al., 1994) Vertebrate RXRα and RXRβ expression both appear early and remain quite broad, with RXRβ expression in all or nearly all cell types including comparatively high expression in the gonads and the brain, and RXRα especially highly expressed in the skin and internal organs. RXRγ expression is typically very low during early development (Mangelsdorf et al., 1992), save for abundant maternal transcripts in frog oocytes that disappear quickly in the early embryo (Blumberg et al., 1992; Wong and Shi, 1995). Developmentally and in adult stages, RXRγ expression is often observed in a more restricted pattern than RXRα or RXRβ, such as in the neural crest, ventral spinal cord, specific brain regions, the anterior pituitary, the retina, and skeletal muscle (Hoover et al., 1998; Mangelsdorf et al., 1992; Rowe and Brickell, 1995). Interestingly, each duplicated zebrafish RXR paralog pair shows overlapping and distinct expression patterns (Waxman and Yelon, 2007b), with one generally similar to mouse and frog ortholog (and thus perhaps ancestral) patterns and the other showing a more divergent pattern, implying potential evolution toward novel and further fine-tuned RXR-NR regulated response pathways (Tallafuss et al., 2006).

Genetic approaches in mice to examine RXR isotype function revealed both overlapping and distinct roles development. Homozygous RXRα null mice die between embryonic stages E13.5 and E16.5, and the observed 100% mortality appears to be due largely to heart malformations including myocardial hypoplasia and defective ventricular septation (Gruber et al., 1996; Sucov et al., 1994). These mice also have impaired eye (Thompson et al., 2019) and liver development with significant skin edema. Using a series of crosses with RAR isotype specific knockout lines, RXRα appears to participate in broad range of RAR actions that are generally reminiscent of vitamin A deficiency effects on development (reviewed in (Mark et al., 2006, 2009). Interestingly, the use of isotype specific RXR mutants to probe genetic interactions with other developmentally important NRs has not been as extensive, save for very specific instances (with LXRβ in Sertoli cells (Mascrez et al., 2004b), TRβ in the cochlea and pituitary (Barros et al., 1998; Barros et al., 1998), and PPARs in keratinocytes (Calléja et al., 2006). In addition to an important role in endogenous retinoid signaling, RXRα appears to be the major RAR partner responsible for mediating the teratogenic effects of exogenous retinoid exposure on limb patterning and craniofacial defects (Matt et al., 2003; Nugent et al., 1999; Sucov et al., 1995). Further, cell specific knockout experiments revealed that a) RXRα involvement in heart development is not cell autonomous e.g. not acting within the cardiomyocytes themselves and b) RXRα has specific roles in keratinocytes and epidermal barrier integrity (M. Li et al., 2005), hepatocyte lifespan, function, and regeneration capacity (Imai et al., 2001), and properly controlled hematopoietic stem cell activity (in combination with RXRβ) (Menéndez-Gutiérrez et al., 2023). Additional genetic models were created to probe the relative contributions of the more ligand dependent, C-terminal AF-2 domain versus the more ligand independent N-terminal AF-1 domain to RXRα function during development, both together and independently. When RXRα AF-1 and AF-2 are missing, mice expressing the transcriptionally silent and truncated RXRα do not show the cardiac defects seen in RXRα null mice but still have eye malformations and late placentation defects (Mascrez et al., 2009). Mice lacking RXRα AF-2 or AF-1 alone show synergistic effects with RAR isotype mutants like the broad spectrum of vitamin A deficiency effects on development (Mascrez et al., 2001). AF-2 appears to be most important here while AF-1 may play more specific roles such as in interdigital mesenchymal remodeling (Mascrez et al., 1998). In all, these results suggest an interesting and complex role for RXR transcriptional activity and endogenous ligand binding in cell and stage specificity within RA mediated developmental pathways, and potentially in response to exogenous ligand disruption.

In terms of specific roles for the other two RXR isotypes, RXRβ null mice survive to adulthood despite its widespread early expression, although a significant number of embryos die perinatally. The most significant adult phenotype is male infertility due to abnormal spermatogenesis such that by adulthood very few or no mature spermatozoa are found in the seminiferous tubules and epididymis (Kastner et al., 1996). In the testes, RXRβ is specifically expressed in Sertoli cells, and lack of functional RXRβ leads to accumulation of cholesterol ester droplets in these cells. In this case, the RXRβ null phenotype is distinct from that of RARα and RARγ null mice or vitamin A deficiency (Gely-Pernot et al., 2012; Vernet et al., 2006), which also lead to male infertility albeit via broader effects on testicular degeneration and sperm maturation (Ghyselinck et al., 2006). Interestingly, generation of mice with RXRβ alleles lacking AF-2, akin to the approach used to probe ligand dependent and independent functions of RXRα, show normal spermatogenesis but still have altered Sertoli cell cholesterol metabolism, a role seemingly mediated via RXRβ/LXRβ heterodimers (Mascrez et al., 2004a). RXRγ knockout mice of both sexes appear healthy and fertile, and offspring of heterozygous mutant crosses showed expected Mendelian ratios. In adult RXRγ null mice, circulating TSH and T4 levels are elevated as are their basal metabolic rates (Brown et al., 2000), although the former was not as significantly changed in other studies (Barros et al., 1998). TSH secretion is partly resistant to exogenous T3 administration RXRγ null mice, again consistent with a role for RXRγ in HPT axis negative feedback, albeit milder than observed in TRβ knockout mice (Abel et al., 2001; Forrest et al., 1996). Nevertheless, it has long been known that RXR ligand treatment results in significant hypothyroidism in mice and humans (Janssen et al., 2011) and 9-cis retinoic acid is less effective in suppressing TSH in thyrotrope derived cell lines with reduced RXRγ expression. In the developing retina, lack of RXRγ leads to an aberrant increase in short wave S-cones (Roberts et al., 2005), similar to what is observed in TRβ2 mutant mice although the latter also show greatly reduced longer wave M-cones (Ng et al., 2001) that is not observed in RXRγ mutants. While neural development appears generally normal in RXRγ null mice, they do show significantly impaired neural remyelination and delayed oligodendrocyte differentiation in models of multiple sclerosis (Huang et al., 2011) as well as a range of affective behavior deficits (Krezel et al., 1996). Finally, through extensive crossing of RXR mutant lines, it appears that only a single functional RXRα allele (RXRα +/−) is absolutely required to support embryo viability in RXRβ and RXRγ null backgrounds (Krezel et al., 1996).

### 2.6. Amphibian RXRs: actions and disruption in metamorphosis

A key component of vertebrate development is a period of high thyroid hormone (TH) activity at the transition from fetal/larval development to juvenile stages (Buchholz, 2015; Sachs and Buchholz, 2017). In amphibian frog development, this is metamorphosis, the process through which tadpoles develop into juvenile frogs (Furlow and Neff, 2006). TH signaling is required to initiate and maintain metamorphosis (Puzianowska-Kuznicka et al., 1997; Schreiber et al., 2001; Yaoita and Brown, 1990). In humans the perinatal TH surge does not result in such dramatic anatomic changes, but it is nevertheless crucial for gut development (Frau et al., 2017; Plateroti et al., 1999) and proper brain and sensory nervous system maturation (Bernal, 2017, 2018; Flamant et al., 2017; Ng et al., 2013; Picou et al., 2012; Richard and Flamant, 2018; Swaroop et al., 2010). In all vertebrates, the molecular mechanisms governing TH action are highly conserved: THs are identical across all taxa, with T3 being the active form; the TRs are highly conserved, and there are two isoforms, TRα and TRβ; they bind corepressors in the absence of TH and coactivators when TH is present; and TRs require RXRs for efficient DNA binding and activation by TH (reviewed in (Brent, 2012; Mendoza and Hollenberg, 2017; Vella and Hollenberg, 2017).

Most work on amphibian RXRs has been conducted using the diploid frog *Xenopus tropicalis* and its allotetraploid cousin *Xenopus laevis*. In the latter, all three RXR encoding genes are duplicated and full-length transcripts are detectable from both subgenome alloalleles (Fisher et al., 2023). The RXR isotype expression patterns in *Xenopus* during embryonic development and in adults are generally similar to other vertebrates. Interestingly, in developing tadpoles, RXRα expression gradually rises post-hatching in parallel to rising levels of TRα, prior to the appearance of circulating thyroid hormones that drive metamorphosis. In particular, RXRα and TRα expression is high in limb buds along with the Type II deiodinase (D2) that converts T4 to T3 at the start of metamorphosis (Cai and Brown, 2004; Wong and Shi, 1995). By contrast, expression levels of TRα, RXRα and D2 are relatively low in the less sensitive tadpole tail, until metamorphic climax when limb development is complete and tail resorption can finally commence when TRβ expression is fully induced at peak TH levels. Xenopus RXRs appear to be biochemically indistinguishable from their mammalian counterparts in terms of TR and RAR heterodimerization potential and the ability to enhance transactivation by T3 bound TRs at TREs and RA bound RARs at RAREs in a chromatin context (Minucci et al., 1998; Wong and Shi, 1995). The ability of forced early expression of TRα in early *Xenopus laevis* embryos to precociously induce TH responsive genes strongly depends on co-expression of RXRα (Puzianowska-Kuznicka et al., 1997). Suggests that RXRs are important limiting factors for the acquisition of TH competence in developing tadpoles. Thus, given the degree of sequence conservation, general expression patterns, NR heterodimerization, and RXR ligand binding properties, investigating *Xenopus* RXR action during development presents an attractive and accessible vertebrate model for addressing important questions about these enigmatic yet developmentally and physiologically important members of the NR superfamily.

As discussed, the TR-RXR heterodimer has been considered non-permissive, and therefore, ligands for RXR would have no role in TR signaling. The RXR was often described as a “silent partner” to the TR, which seemed to make sense with the finely regulated role of TH in metabolic homeostasis and the high affinity of T3 for TRs (Evans and Mangelsdorf, 2014). We created the GH3. TRE-Luc cell line, by integrating a 2X-canonical DR4 thyroid hormone response element (TRE) that drives firefly luciferase expression in the presence of T3 into the GH3 rat pituitary cell line that expresses all TH-binding forms of TRs (i. e., TRα, TRβ1, TRβ2) (Freitas et al., 2011, 2014). These cells were used to assess the ability of chemicals to disrupt TH signaling in the Tox21 program (https://tox21.gov/), a collaboration of the United States Environmental Protection Agency (US-EPA), the National Institute of Environmental Health Sciences, the National Center for Advancing Translational Sciences, and the Food and Drug Administration. However, the positive hits, outside of the natural and synthetic versions of the THs, were mostly ligands for RXRs. We verified that RXR retinoids and designed agonists did activate the TRE-Luc and endogenous TR-target genes like growth hormone; further, an RXR antagonist inhibited RXR agonist-driven TRE-Luc activity (Mengeling and Furlow, 2015). In addition to natural retinoids and RXR pharmaceuticals, we also examined the pollutants TBT, already shown to cause imposex in mollusks through RXR, and triphenyltin (TPT), a fungicide still in use in the US on crops such as beets, potatoes, and pecans. These organotins were shown to function through RXR to suppress B cell development in bone marrow, and to affect adiposity in offspring of female mice exposed to TBT during pregnancy (Baker et al., 2017; Chamorro-García et al., 2013; X. Li et al., 2011). TBT and TPT covalently bind to a Cys residue at the mouth of the ligand binding pocket of RXRs and trigger an active form of the RXR ligand binding domain, while not inhabiting the binding pocket itself (le Maire et al., 2009; Nakanishi et al., 2005). Trimethyltin (TMT), in comparison, can covalently bind to the Cys, but the methyl groups are too small to activate the RXR. We found that both TBT and TPT, but not the inactive trimethyltin (TMT), induced the TRE-Luc reporter in the GH3. TRE-Luc cells at nanomolar concentrations (Mengeling et al., 2016). These cellular results prompted us to test the ability of organotins to effect TH signaling in the developing tadpole.

**Fig. 4.**
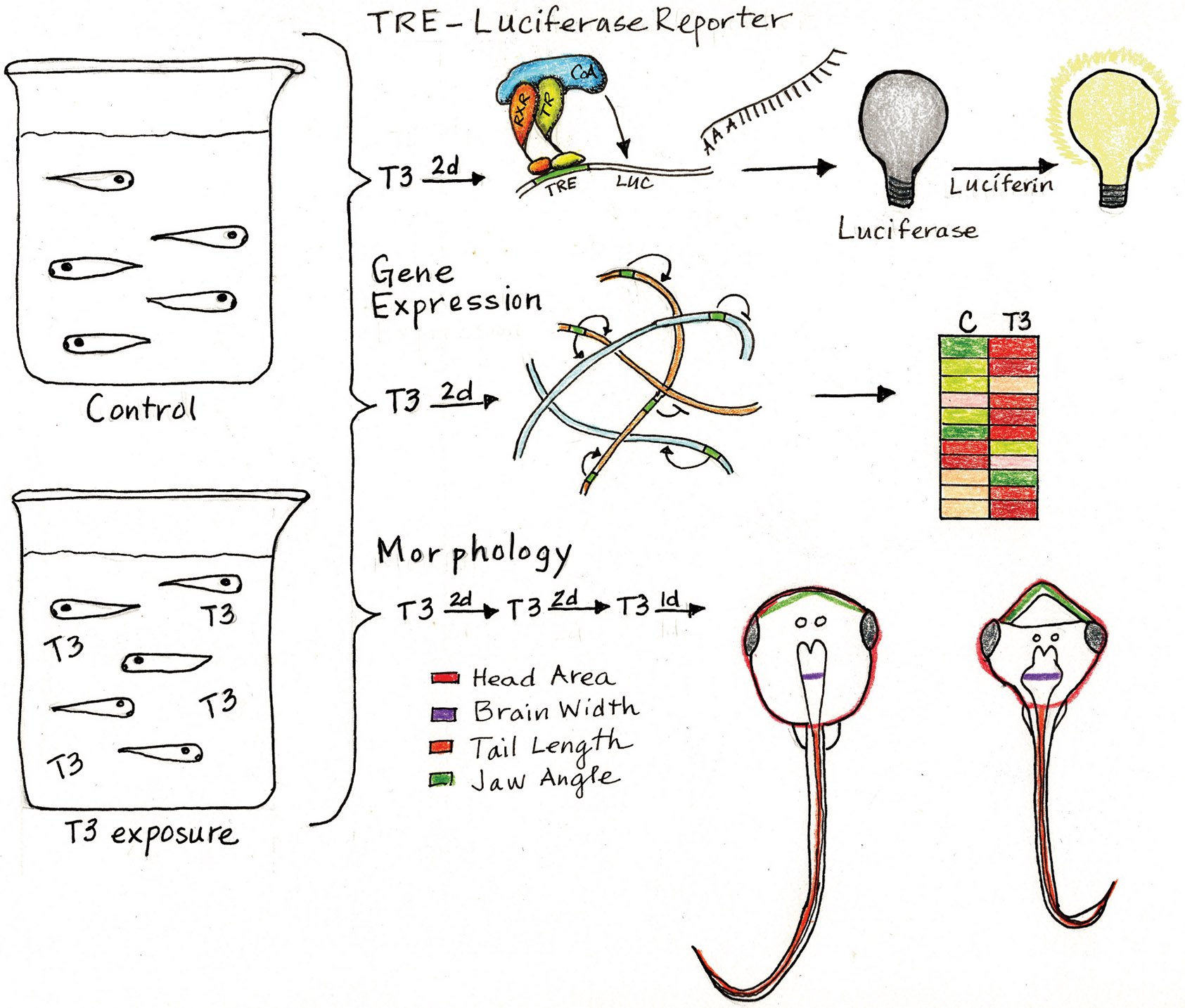
The *Xenopus laevis* “One-Week Assay” uses tadpoles at one-week post-fertilization to assess the effects of exogenous TH exposure in the presence and absence of potential disruptors of TR and/or RXR on TH signaling through three main assays. Transgenic tadpoles with an integrated luciferase reporter gene under the control of T3 through a TRE are assayed for luciferase activation after two days of treatment. Differential gene expression after two days of treatment can be interrogated through transcriptomics or RT-qPCR on different body parts, such as tail, brain or lower jaw. Morphological changes follow gene expression changes, and after five days of treatment are quantifiable. T3 triggers metamorphic gene expression profiles resulting in lower jaw and brain remodeling and gill resorption, which is measured by changes in head area, and tail resorption, which shortens the tail.

At one-week post-fertilization, *Xenopus laevis* tadpoles enter an approximately two-week isometric growth period, during which they increase in size without further development. At the start of this period, they have not yet been feeding, and they are very uniform in size. Tadpoles can withstand nine days post-depletion of the yolk sac without feeding (McKeown et al., 2017; McKeown and Cline, 2019), although growth will not commence until feeding starts. Since tadpoles at this stage have not yet developed a thyroid gland, they have no circulating THs; however, they do express TRα, which has been shown to repress genes involved in limb development from premature activation (Buchholz and Shi, 2018; Tanizaki et al., 2022; Wen et al., 2017; Wen and Shi, 2015, 2016). Addition of exogenous T3 to the rearing water of these young tadpoles activates many metamorphic genetic programs precociously, and we took advantage of that to develop a suite of assays (Fig. 4), including morphological measurements and an integrated TRE-driven luciferase reporter gene, to assess the ability of man-made chemicals to disrupt TH signaling (Mengeling et al., 2017).

When we exposed young tadpoles to TBT or TPT in the absence of T3, their morphologies were identical to vehicle-treated controls, and the TRE-Luc gene was not activated, which was different than in the GH3. TRE-Luc where the TBT and TPT could activate the reporter in the absence of T3. However, when we co-exposed the tadpoles to TBT or TPT in the presence of T3, TBT and TPT potentiated the effect of T3 to the extent that a 5 mM T3 + 1 nM TBT or TPT exposure was the equivalent of a 25 nM T3-only exposure (Mengeling et al., 2016). This was especially noticeable in lower jaw remodeling and tail resorption (Mengeling et al., 2016, 2022), both of which normally occur late in the metamorphic process (tail resorption is the last step of metamorphosis). The data suggest that the organotins were functioning as competence factors for TH signaling, increasing the ability of the tadpole to respond to T3. We then compared the ability of TBT to potentiate T3 signaling to the pharmaceutical RXR agonist bexarotene (Bex) and the designed RXR antagonist UVI 3003. Bex co-exposure with T3, like TBT and TPT, potentiated morphological changes and induction of the TRE-Luc reporter, further supporting that the organotins were functioning through RXR. Furthermore, UVI 3003 co-exposure with T3, completely abrogated the T3 response at morphological and reporter level (Mengeling et al., 2018), which is in line with data indicating that RXRα is required for metamorphosis (Puzianowska-Kuznicka et al., 1997). At the transcriptomic level in the resorbing tail, there was an 80% overlap between T3-regulated genes co-exposed to Bex or TBT, and the direction of regulation (activated or repressed) were the same between both groups. For the morphological assays and reporter, 30 nM Bex provided the maximum response, and in the transcriptomic analysis 30 nM Bex affected only 130 genes on its own compared to increasing the significant number of T3-regulated genes from 3200 to 6700 (Mengeling et al., 2018), indicating the large increase in T3 competence mediated by RXR agonism at this early developmental stage. Extracellular matrix metalloproteinase expression and transcription factors were major classes of genes TBT and Bex potentiated T3-induced regulation. The above results suggest that inappropriate RXR ligand exposure can disrupt TH-controlled developmental programs by changing the competence for T3 signaling. We found that in pro-metamorphic tadpoles (NF 54), which are considered fully competent for responding to T3, the TRE-reporter in the lower jaw was still potentiated by co-exposure to Bex (Mengeling et al., 2022). This is a stage before normal jaw remodeling commences, suggesting that control of RXR liganding may be a mechanism through which the tadpole controls developmental timing, and highlight the importance of both dose and timing to proper development.

Recently, we used an in silico machine learning approach (Ramaprasad et al., 2022) to probe a chemical database of over 57,000 chemicals (Mansouri et al., 2020) searching for other chemicals with potential to disrupt RXR signaling. Several tert-butyl phenols were positive hits, and we tested several in our in vivo precocious tadpole-based TRE-Luc and morphology assays. 2,4,6-tri-tert-butylphenol potentiated T3 action in the tapoles at 30 nM (Mengeling et al., 2025). Replacing moieties at the C4 position showed that a 4-secbutyl group was nearly as effective as the tert-butyl, while a 4-ethyl group was the smallest sized group we tested that still potentiated at concentrations less than 1 μM (Mengeling et al., 2025). These compounds are used in food contact materials as antioxidants (Ji et al., 2024; Liu et al., 2017; Liu and Mabury, 2019, 2020), including in the coating on baby bibs, hot beverage containers, and microwavable food boxes. Our findings raise concerns over ingestion of potential endocrine disrupting compounds that can affect the action of RXR, which again is a binding partner for nearly a third of all NRs. Our findings highlight the need for further screening of the millions of man-made chemicals present in items people are exposed to on a daily basis for their ability to disrupt RXR function.

### 2.7. Future directions

RXRs are among the most ancient and most conserved of the nuclear receptors. They have evolved to be the required binding partner for many other NRs that are necessary for animal development, reproduction, and metabolism. No one has found a vertebrate cell type without any RXR expression. While this ubiquity speaks to the fundamental importance of RXRs for NR gene expression programs, it also creates complexity. The advent of single-cell and single gene resolution assays for both gene expression and chromatin landscape changes can now begin to address this complexity. Further, gene-editing techniques can further probe the advantages gained by expansion of RXR from one protein to three isoforms in the vertebrate lineage. In addition, in situ gene-editing could allow expression of RXR mutant alleles expected to change self-oligomerization, heterodimerization, or post-translational modifications to determine molecular mechanisms of gene regulation beyond agonist and DNA binding. Comparative analysis across animal phyla representing evolutionary time can help us understand how evolution affects ligands that bind RXR. The combined application of these approaches can then be used to deeply interrogate exogenous chemicals for RXR disruption with the goal of preventing adverse biological outcomes in both our environment and human health.

## Data Availability

No data was used for the research described in the article.
